# Supplementary material for: Antibiotic Utilization Patterns for Different Wound Types among Surgical Patients: Findings and Implications
Source: Antibiotics (Basel). 2023 Mar 30;12(4):678. doi: 10.3390/antibiotics12040678 (PMC10135394; doi:10.3390/antibiotics12040678)
Supplement: Supplementary file 1 [file antibiotics-12-00678-s001.zip › antibiotics-2284090-supplementary.pdf]

Supplementary Tables

Table S1. Length of prescribing of antibiotics post-operatively among a range of LMICs to prevent SSIs

**Low Middle-Income Countries***	
Burkina Faso - 2019 [79]	• Prolonged administration of antibiotics for SAP was common<br>• Administration for > 2 days in 87.1% of cases
Congo - 2020 [80]	• 69.1% of patients were prescribed antibiotics for 3 days or longer post operatively<br>• Ampicillin was the most frequent antibiotic prescribed (43.8% of patients) followed by cloxacillin (13.2%), and gentamicin (9.4%)
Egypt – 2022 [81]	• 98.5% of patients (134/136) were prescribed antibiotics for > 24 hours for SAP; median duration was 3 days (range, 2–9 days)<br>• No patient was prescribed a single-dose prophylaxis pre-operatively
Ethiopia - 2018 [44]	• 79.1% of surveyed SAP patients were prescribed antibiotics for 2 days or more, with 34.7% prescribed antibiotics for > 5 days<br>• Approximately 84% of the patients were prescribed ceftriaxone either alone or in combination for SAP
Ethiopia - 2022 [82]	• 82.6% had antibiotics prescribed for >1 day to prevent SSIs, with the average number of antibiotics prescribed per patient at 1.32<br>• Ceftriaxone (54.7% of antibiotics) was a widely prescribed antibiotic for SAP
Ghana - 2019 [83]	• 88.4% of patients were prescribed antibiotics for > 1 day to prevent SSIs, with 9.9% of patients prescribed SAP for one day and only 1.6% receiving a single dose of antibiotics to prevent SSIs<br>• Most commonly prescribed antibiotics for SAP were cephalosporins (28.9% of patients) and co-amoxiclav (28.1%)
Ghana - 2021 [84]	• 75.5% of antibiotics prescribed for SAP were for > 1 day
Ghana - 2022 [11]	• The duration of SAP among patients surveyed was 6.9± 2.1 days<br>• Most common antibiotics prescribed were a combination of cefuroxime and metronidazole (56.1%, *n*=335) followed by co-amoxiclav (14.2%)
Kenya - 2018 [85]	• The average number of doses of antibiotics for SAP among surveyed patients was 19.1<br>• Most frequently prescribed antibiotics on the surgical wards (most for SAP) were third generation cephalosporins
Kenya - 2019 [86]	• 76.9% of surveyed patients were prescribed SAP for > 1 day<br>• Only 9.6% of surveyed patients were administered a single dose of antibiotics for SAP
Nigeria - 2017 [87]	• Antibiotic prescriptions for SAP were given for >1 day in 95.0% of patients undergoing surgery

	• Ceftriaxone (28.0% of situations), metronidazole (20.0%) and cefuroxime (17.0%) were the most prescribed antibiotics for SAP
Nigeria - 2021 [88]	• 94.8% patients had antibiotics administered for SAP for longer than 24 hours; with 4.2% of patients administered for one day and only 1.0% of patients prescribed one dose for SAP<br>• Metronidazole, cefuroxime, ceftriaxone and ciprofloxacin were the most prescribed antibiotics in the surgical wards
Nigeria - 2022 [89]	• All surveyed patients were prescribed antibiotics for SAP post-operatively<br>• For 24 hours in 23.8% of patients and > 1 day in 76.2% of patients
Rwanda - 2019 [90]	• Nearly all women undergoing SAP had antibiotics prescribed post operatively to reduce SSIs
Tanzania - 2020 [91]	• Out of 57 patients, 33% had antibiotics prescribed for SAP for 2 – 3 days and 56% for > 3 days<br>• Ceftriaxone was the most prescribed antibiotic for SAP ($n = 28$; 49%)
Tanzania - 2021 [84]	• 97% of patients undergoing surgery had antibiotics prescribed for SAP for > 1 day
Tunisia – 2019 [92]	• Typical duration of SAP was >1 day
Uganda - 2021 [84]	• 97.1% of antibiotics that were prescribed for SAP were prescribed for > 1 day
Uganda - 2022 [93]	• 98.4% of patients had multiple doses of antibiotics for SAP for > 1 day (301 patients overall)<br>• Ceftriaxone and metronidazole were the principal antibiotics prescribed for SAP
Zambia - 2021 [84]	• 96.5% of patients undergoing surgery had antibiotics prescribed for SAP for > 1 day (83 patients overall)
**Upper Middle-income Countries***	
Botswana - 2018 [13]	• Prolonged administration for SAP was common with a mean (SD) duration of 5 (+/- 2.6) days, greatest for emergency surgery (72.7% of surveyed patients)<br>• The most commonly prescribed antibiotics were cefotaxime (80.7% of situations) and metronidazole
Iraq – 2018 [94]	• All patients undergoing SAP were prescribed antibiotics >1 day.
Iraq – 2021 [95]	• 65.5% of surgical and medical prophylactic antibiotics were used for > 1 day, especially ceftriaxone
Jordan - 2020 [96]	• 8.7% of patients were prescribed a single dose of antibiotics for SAP, 30.4% more than one dose for one day and 60.9% > 1 day
South Africa - 2021 [97]	• In 73.2% of cases, SAP were prescribed antibiotics for > 1 day<br>• Cefazolin was the most commonly prescribed antimicrobial (45.5% of cases) for SAP followed by co-amoxiclav (22.3% of cases)
South Africa - 2022 [98]	• In 66.7% of pediatric cases (10 out of 15 patients), antibiotics for SAP were prescribed for > 1 day

NB: ASP: Antimicrobial Stewardship Program; SAP: Surgical Antibiotic Prophylaxis; SSIs: Surgical Site Infections; * World Bank Status (Based on [99])

Table S2. Programs instigated to improve antibiotic prescribing for SAP among LMICs

Country, year and reference	Summary of the intervention	Outcome
4 African countries (Kenya, Uganda, Zambia, and Zimbabwe) 2018 [100]	• Appreciable educational activities including five planned visits to each hospital alongside a range of tools to improve SAP<br>• Local teams identified key areas to concentrate on and identified appropriate indicators<br>• Subsequent monitoring of activities and feedback	• SSIs decreased to 3.8% of operations post interventions (down from 8.0% - p <0·0001)<br>• Appropriate use of SAP improved to 39.1% of patients – up from 12.8% at baseline (p <0·0001)
Egypt – 2015 [101]	• Extensive education including a 2-day training course coupled with posters and on-the-job training<br>• Regular audit and feedback meetings	• All hospitals showed a significant rise in the optimal duration of antibiotics for SAP (p < 0.01)<br>• The optimal timing of the first dose also improved significantly in 3 hospitals to 38.7% of patients (up from 6.7% p < 0.01)
India - 2018 [102]	• Agreement among key stakeholders regarding the key elements of the WHO checklist to help reduce SSIs<br>• The designated checklist coordinator confirmed that the surgical team had completed its tasks before proceeding to the next steps during the operations	• Prior to implementation - all patients operated on a particular day were administered antibiotics in the morning irrespective of the timing of their surgery<br>• Following the intervention - the correct practice of administration within 1 to 2 hours of the incision became normal practice<br>• In addition, addressing concerns that appropriate surgical patients were not given SAP
Iran - 2019 [103]	• SAP guidelines revised following meetings between pharmacists and the surgical department following lectures and discussion concerning SAP to key members of the surgical departments<br>• Clinical pharmacists participating in ward rounds, communicating with surgeons when guidelines not followed and providing educational material on SAP<br>• Rationality of SAP continually evaluated in accordance with agreed guidelines with clinical pharmacists communicated any concerns directly with relevant physicians	• Rate of antibiotic prescribing beyond 48 hours decreased from 92.1% of patients to 5.7% of patients post intervention<br>• The appropriateness of antibiotic use increased from 30.1% to 91.4% of patients<br>• The mean cost of antibiotics decreased more than 11-fold and length of stay from an average of 5.14 days to 4.33 days (p<0.001)
Kenya - 2013 [25]	• Appreciable educational activities including developing an SAP guideline<br>• Subsequently monitoring adherence to agreed guidelines	• Significant improvement in reducing post-operative antibiotics for SAP to 60% of patients during week 1 and 90% in week 6 post intervention (p< 0.0001)

		• Net reduction in costs including antibiotics and associated consumables
Kenya - 2015 [104]	• Education, training and leadership programs among key front-line staff<br>• Frequent monitoring of activities post-ASP intervention	• SAP patients prescribed antibiotics post-operatively decreased from 50% to 26% of patients<br>• Crude SSI rates significantly decreased to 5% of patients (down from 9.3%)
Moldova - 2015 [105]	• Introduction of a surgical safety checklists in the operating theatre<br>• The data collection team developed and randomly assigned to observe 30% of the surgical cases, appraise adherence to agreed practices as well as feedback the findings	12.7% increase in the appropriate use of antibiotics for SAP
Nigeria - 2019 [106]	• Extensive educational activities including development and dissemination of agreed SAP protocol combined with regular meetings with key clinicians and reminders on all-mounted posters<br>• Regular audit and feedback meetings	• SAP patients in the post-intervention period were 5.6 times more likely to receive antibiotics within 60 minutes before the incision vs. pre-intervention ($p < 0.001$)<br>• Redundant antibiotic prescriptions were reduced by 19.1%
Pakistan – 2019 [29]	• Educational interventions including sharing the findings in the pre-intervention arm with all key stakeholders alongside discussions on appropriate SAP<br>• Educational training continued for 10–15 days<br>• Post-intervention - data collected and shared to improve future SAP	• Compliance of correct antibiotic choice, dose, frequency and duration increased from 1.3% to 12.4%<br>• Significant reduction in the mean duration of SAP (17%, $p = 0.003$), average number of prescribed antibiotics (9.1%, $p = 0.014$) and average antibiotic cost (25.7%, $p = 0.03$)<br>• Reduction in hospital costs ($p = 0.003$) and length of stay ($p = 0.023$).
Pakistan – 2020 [55]	• Conducted an audit of antibiotic prophylaxis in surgical procedures to evaluate adherence to pre-operative antibiotic prophylaxis (PAP) practices.<br>• Investigated the three most commonly performed elective general surgical procedures in adults aged > 18 years with no previous infection or surgery.<br>• Recommended training and awareness programs about antimicrobial stewardship interventions to improve adherence to guidelines.	• PAP was given in most cases, but the choice of antibiotics was correct in only a small percentage of patients based on existing guidelines.<br>• Compliance to the timing of antibiotics was low.<br>• Non-use of PAP was observed in a significant percentage of cases.
Pakistan – 2021 [107]	• The intervention was a randomized, double-blind placebo trial.	• The study aimed to compare the efficacy of post-operative oral antibiotics versus placebo in reducing the frequency of

	• The experimental group received post-operative oral antibiotics following appendectomy for non-perforated appendicitis.<br>• The placebo group did not receive post-operative oral antibiotics following appendectomy for non-perforated appendicitis.	surgical site infection (SSI) following appendectomy for non-perforated appendicitis.<br>• The study found no significant difference between the experimental and placebo groups in terms of frequency of SSI.<br>• The results suggest that post-operative oral antibiotics may not be effective in reducing the frequency of SSI following appendectomy for non-perforated appendicitis.
South Africa - 2017 [108]	• SAP 'toolkit' alongside regional training and institutional workshops<br>• Consensus and endorsement from key groups for SAP guidelines and modifying guidelines where appropriate<br>• Compliance to SAP guidelines measured alongside feedback to improve future compliance	• Timely administration of antibiotics for SAP increased to 56.4% of surgical patients ($P < 0.0001$)<br>• Optimal duration of antibiotic administration for SAP increased to 93.9% of patients
Turkey – 2014 [109]	• A number of educational activities were instigated including a series of meetings with physicians from each clinic<br>• Alongside this, daily visits from the Infection Control Nurse as well as regular visits (twice per week) from an Infectious Diseases Control Specialist – more if compliance with agreed guidelines was low<br>• Activities and outcomes regularly shared with the physicians	• Prescribing of appropriate antibiotics for SAP increased from 51% to 63.4% of cases<br>• Duration of prescribing improved from 10.3% to 59.4% of cases<br>• Total cost of antibiotics in surgical units decreased by 38.6%
Turkey – 2019 [110]	• Local guidelines updated by two members of the infection control committee with one surgery team leader responsible for improving SAP as part of ASPs in their group<br>• Periodic training sessions - supervised and regulated by the SAP surgical team leaders<br>• Agreed that clean and clean-contaminated cases would not be given SAP for longer than 24 hours and that discharge prescriptions would not include antibiotics	• Compliance with the indication of SAP increased from 55.6% to 64.5% of patients ($p<0.05$)<br>• Significant impact on antibiotic prophylaxis beyond 24 hours – reducing from 60.2% of patients before the intervention to 7.5% after ($p<0.05$)<br>• Extent of antibiotic prescribing after discharge reduced from 80.6% of patients to 9.4% ($p<0.05$)<br>• Limited impact on the timing of the first antibiotic dose – appropriate timing increased from 81.9% of patients to 83.7%
